# Immunogenicity and Safety of Chikungunya Vaccines: A Systematic Review and Meta-Analysis

**DOI:** 10.3390/vaccines12090969

**Published:** 2024-08-27

**Authors:** Annalisa Rosso, Maria Elena Flacco, Giovanni Cioni, Marco Tiseo, Gianmarco Imperiali, Alessandro Bianconi, Matteo Fiore, Giovanna Letizia Calò, Vittorio Orazi, Anastasia Troia, Lamberto Manzoli

**Affiliations:** 1 School of Public Health, Department of Environmental and Prevention Sciences, University of Ferrara, Via Fossato di Mortara 44, 44121 Ferrara, Italy; annalisa.rosso@unife.it (A.R.); mariaelena.flacco@unife.it (M.E.F.); giovanni01.cioni@edu.unife.it (G.C.); marco.tiseo@edu.unife.it (M.T.); gianmarco.imperiali@edu.unife.it (G.I.); giovannaletizia.calo@edu.unife.it (G.L.C.); vittorio.orazi@edu.unife.it (V.O.); anastasia.troia@edu.unife.it (A.T.); 2 School of Public Health, Department of Medical and Surgical Sciences, University of Bologna, Via San Giacomo 12, 40126 Bologna, Italy; alessandro.bianconi4@studio.unibo.it (A.B.); matteo.fiore7@studio.unibo.it (M.F.)

**Keywords:** chikungunya fever, chikungunya vaccine, VRC-CHKVLP059-00-VP, VLA1553, MV-CHIK, immunogenicity, vaccine safety, meta-analysis

## Abstract

Several vaccines against chikungunya fever have been developed and tested, and one has been recently licensed. We performed a meta-analysis to estimate the immunogenicity and safety of all chikungunya vaccines that have been progressed to clinical trial evaluation (VLA1553; mRNA-1388/VAL-181388; PXVX0317/VRC-CHKVLP059-00-VP; ChAdOx1 Chik; MV-CHIK). We included trials retrieved from MedLine, Scopus, and ClinicalTrials.gov. The outcomes were the rates of seroconversion/seroresponse and serious adverse events (SAEs) after the primary immunization course. We retrieved a total of 14 datasets, including >4000 participants. All candidate chikungunya vaccines were able to elicit an immunogenic response in ≥96% of vaccinated subjects, regardless of the vaccination schedule and platform used, and the seroconversion/seroresponse rates remained high 6 to 12 months after vaccination for most vaccines. Four of the five candidate vaccines showed a good overall safety profile (no data were available for ChAdOx1 Chik), with no significant increase in the risk of SAEs among the vaccinated, and a low absolute risk of product-related SAEs. Overall, the present findings support the potential use of the candidate vaccines for the prevention of chikungunya and the current indication for use in adult travelers to endemic regions of the licensed VLA 1553 vaccine. In order to extend chikungunya vaccination to a wider audience, further studies are needed on individuals from endemic countries and frail populations.

## 1. Introduction

Chikungunya fever is caused by an alphavirus (Chikungunya virus—CHIKV) vectored to humans by daytime-biting *Aedes* mosquitoes, and has been endemic for decades in several African and Asian countries [1]. Approximately 40% of the infected individuals experience chronic symptoms, and 0.3% become deceased [2,3]. In the last two decades, due to the expansion of *Aedes* mosquitos to new environments [4,5], CHIKV autochthonous transmission has been documented in 114 countries, with over 5 million cases reported globally [4]. Currently, three quarters of the world’s population are estimated to be living in areas at-risk of viral transmission [6], and the spread of CHIKV has been recognized as a major global threat to public health [7].

There is no antiviral drug available for chikungunya [8], and prevention strategies have been based predominantly on mosquito population control [8,9], which has proved to be challenging [8,10]. One vaccine (VLA1553) has been recently licensed by the Food and Drugs Administration (FDA) for the travelers to endemic areas [11] and was recommended for marketing authorization in the European Union by the European Medical Agency (EMA) [12]. In addition, several other vaccines have been developed and tested, progressing through preclinical and clinical stages [8,13].

Data from single trials on single vaccines are fragmented and heterogeneous, and no systematic review has been published to summarize the state of the art of the various vaccines that have been tested so far. As such, we carried out a systematic review and meta-analysis to estimate the immunogenicity and safety of all chikungunya vaccines that have been progressed to clinical trial evaluation.

## 2. Materials and Methods

### 2.1. Search Strategy, Selection Criteria and Methodological Quality

The reporting of this meta-analysis was guided by the standards of the Preferred Reporting Items for Systematic Review and Meta-Analysis (PRISMA) Statement [14]. We extracted data from clinical trials (either randomized or single-arm) evaluating the immunogenicity and/or the safety of various chikungunya vaccines among subjects of all ages. One single group of investigators (A.B., A.T., G.L.C., G.C., M.F., G.I., M.T., and V.O.) searched MedLine, Scopus, and ClinicalTrials.gov using various combinations of the following search terms: “chikungunya OR chikungunya virus OR CHIKV” AND “vaccin*” AND “trial*” (last search update 6 June 2024). While maintaining a common overall architecture, several alternative strings were used after adjustment for each database. The reference lists of reviews and retrieved articles were also searched, and no language or date restrictions were used. In cases of multiple publications focusing on the same population, we decided to include the most recent or the one with the longest follow-up. The list of articles excluded after the full-text screening process and the reasons for the exclusion have been reported in the online Table S1. The methodological quality of the included studies was assessed using the revised Cochrane risk of bias tool [15]. Discrepancies in study selection and/or quality assessment were solved by a senior author (L.M.).

### 2.2. Primary Outcome: Immunogenicity

We analyzed five different chikungunya vaccines: (1) Live-attenuated VLA1553 [16]; (2) mRNA-based mRNA-1388/VAL-181388 [17]; (3) Virus-like particle vaccine PXVX0317/VRC-CHKVLP059-00-VP [18]; (4) Adenovirus vectored ChAdOx1 Chik [19]; and (5) Live-attenuated, recombinant, measles-vectored vaccine MV-CHIK [20] (Table S2). As Chikungunya virus epidemiology and outbreaks are unpredictable, and efficacy trials are considered unfeasible [4], all the extracted trials used immunogenicity outcomes as surrogates of efficacy, in agreement with FDA and EMA approach [21]. Accordingly, the primary outcome was the rate of seroconversion or seroresponse after the primary course of vaccination (one or two doses depending on the vaccine type). Seroresponse/seroconversion rates were extracted from single studies following the definition provided by the authors (Table S2). When present, the control group included subjects who received either placebo alone or placebo in combination with live attenuated MMR vaccine.

### 2.3. Secondary Outcomes: Serious Adverse Events

In order to assess vaccine safety, we considered only serious adverse events (SAEs), defined as life-threatening events or events resulting in persistent disability, hospital admission, or death, and coded according to the Medical Dictionary for Regulatory Activities. We extracted and separately analyzed the results of any SAEs, either related or unrelated to the study product by the investigators, and only the SAEs that were considered related to the administered product. SAEs data were extracted from the first day after the first dose up to the longest follow-up available.

### 2.4. Data Analysis

We first performed meta-analyses of proportions, combining: (a) the seroconversion/seroresponse rates of vaccinated individuals 28 days after one or two vaccine doses (according to the specific vaccine primary course; short-term seroconversion/seroresponse); (b) the seroconversion/seroresponse rates of vaccinated individuals 28 days after one or two vaccine doses 6–12 months after one or two vaccine doses (long-term seroconversion/seroresponse; (c) the rates of any SAEs; (d) the rates of product-related SAEs. Also, we compared the risk of SAEs of the vaccinated subjects versus controls using random-effect head-to-head meta-analyses. All immunogenicity and safety analyses were performed using Per-Protocol (PP) data and were stratified by vaccine type and number of doses. The results were expressed as risk ratio (RR) and 95% confidence interval (CI) and the statistical heterogeneity was quantified using the I2 metric. We used Stata version 13.1 (Stata Corp., College Station, TX, USA, 2013) and RevMan 5.4 (The Nordic Cochrane Centre, The Cochrane Collaboration, 2020, Copenhagen, Denmark) to perform proportion and head-to-head meta-analyses, respectively.

## 3. Results

### 3.1. Characteristics of Included Studies

**Figure 1:**
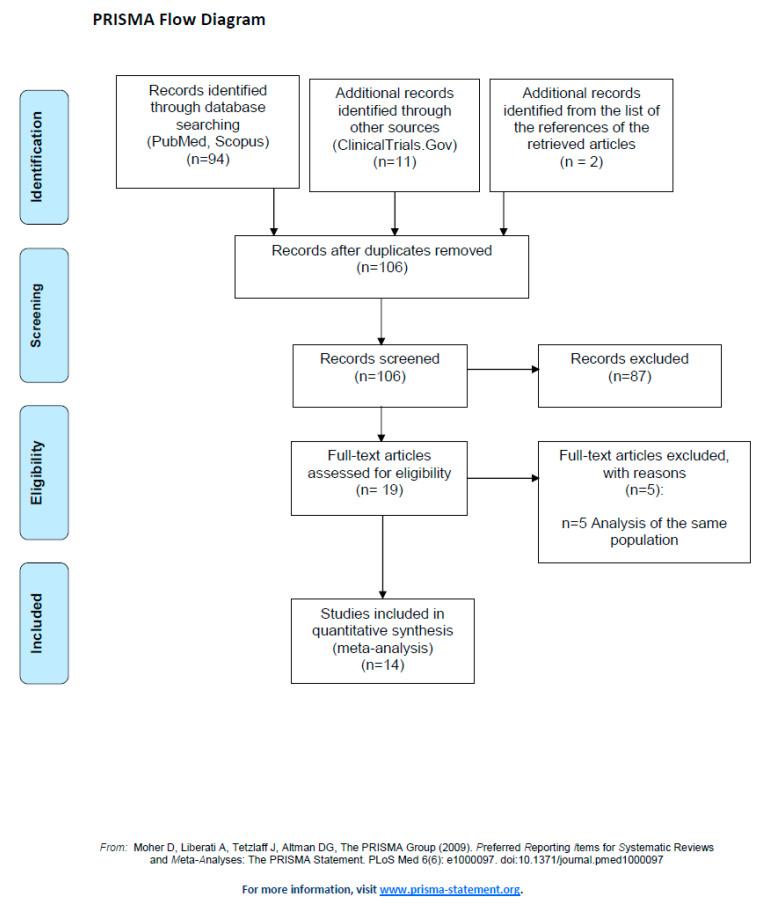
PRISMA 2009 Flow Diagram [14].

From the 107 screened records (Figure 1), we selected 4 ClinicalTrials.gov reports [22,23,24,25] and 10 papers [16,17,18,19,20,26,27,28,29,30], including 14 trials evaluating the immunogenicity and/or the safety of various chikungunya vaccines among the adults (age at enrolment: 18–65 years). Overall, three were single-arm trials [18,19,25] and eleven were classified as RCTs [16,17,20,22,23,24,26,27,28,29,30]. However, four of the RCTs were included in the analyses as single-arm trials [16,23,26,28] as they did not compare vaccinated versus unvaccinated subjects (e.g., dose-finding RCTs). One trial was considered an RCT for the safety analyses and a single-arm trial for the evaluation of immunogenicity, as seroconversion data were not available for the unvaccinated individuals [20]. As reported in Table 1, immunogenicity data could be extracted from eleven studies, and safety data were available from the fourteen included trials.

The main characteristics and the methodological quality of the included studies are reported in Table 1 and Table S3, respectively. Five studies evaluated the immunogenicity and/or safety of MV-CHIK [20,22,23,24,29]; four studies used the virus-like particle vaccine PXVX0317/VRC-CHKVLP059-00-VP [18,25,26,27]; three trials administered the live-attenuated VLA1553 vaccine [16,28,30]; and one trial each evaluated the mRNA-based VAL-181388/mRNA-1388 vaccine [17] and the adenovirus-vectored ChAdOx1 Chik [19]. Seven studies were carried out in the USA, two in UK, two in the European Union, and three in Central American nations, where chikungunya is endemic. Eleven trials were sponsored by the vaccine manufacturer [16,17,20,22,23,24,25,26,28,29,30], one was sponsored by a University fund [19], and two trials received no external funding [18,27]. The number of doses of the primary immunization course varied by vaccine: one dose (ChAdOx1 Chik and VLA1553) or two doses administered one month apart (MV-CHIK, PXVX0317, and mRNA-1388). Finally, the five vaccines also differed by DENV strain: PXVX0317 and mRNA-1388 were based on the Senegal strain 37,997 (West African genotype), VLA1553 and MV-CHIK on the La Reunion strain (East Central South African genotype), and ChAdOx1 Chik was based on multiple CHIKV lineages (Table S2).

### 3.2. Study Quality

The methodological quality of the 14 included trials has been reported in Table S3. All the RCTs [16,17,20,22,23,24,26,27,28,29,30] showed a low overall risk of bias, as they carried a low risk of bias in each of the items of the Revised Cochrane risk-of-bias tool: randomization process, deviation from intended interventions, missing outcome data, measurement of the outcome, and selection of the reported results. The three single-arm trials [18,19,25], in addition to the randomization process, showed some concerns in the measurement of the outcome.

### 3.3. Immunogenicity-30 Days after the First Dose

Eleven datasets, including a total of 1441 individuals, reported immunogenicity data one month after the primary vaccination course (one or two doses; Table 2). All tested vaccines were able to achieve very high pooled seroconversion/seroresponse rates (≥96.5%) with no difference by number of doses and an overall summary rate of 99.6%.

Seven datasets (n = 1131) evaluated immunogenicity at least six months after vaccination. The pooled seroconversion/seroresponse rates remained stable (≥97.9%) for three vaccines (VLA1553, PXVX0317, and mRNA-1388), declined to 71.0% for the MV-CHIK vaccine (although the analysis was based upon a very scarce sample: n = 93), and no data were available for VAL-181388. Again, the immunogenicity did not substantially vary by number of doses (98.2% after a single dose; 93.1% after two). The forest plots of each proportion meta-analysis evaluating the immunogenicity are shown in Figures S1 and S2.

### 3.4. Safety–Serious Adverse Events

Seven RCTs, including a total of 4898 subjects, compared the rates of any or product-related SAEs among the vaccinated subjects versus controls, and could thus be included in head-to-head meta-analyses (Table 3). No data were available for ChAdOx1 Chik, but all the other vaccines did not significantly increase the risk of any SAE, nor of product-related SAEs. Overall, the pooled relative risks (RRs) of any or product-related SAEs were 0.80 (95% Confidence Interval—CI: 0.38–1.69) and 0.98 (0.37–2.59), respectively.

In addition to the seven RCTs shown above, seven trials reported safety data only for the vaccinated subjects, thus the proportion meta-analyses estimating the overall frequency of SAEs among the vaccinated subjects included a total of fourteen trials (Table 2 and Figures S3 and S4). Overall, during a follow-up that lasted 6–12 months for most trials, a total of 69 SAEs of any sort were reported among 4480 vaccinated subjects (pooled rate 6.9‰; 95% CI: 3.9–10.5‰), with no substantial variation by vaccine type. Although the difference was not significant, the pooled rate of any SAE after a single dose was approximately half the rate after two doses (5.8‰ vs. 11.5‰). Finally, there were a total of four SAEs that were considered related to the vaccine by the investigators, for a raw rate of 0.9‰ and pooled rate of 0.0‰. Again, no significant differences were observed by vaccine type or number of doses. The product-related SAEs consisted of a grade 4 aspartate aminotransferase increase [17], one mild myalgia, and one syndrome of inappropriate antidiuretic hormone secretion [30] and arthritis [29].

### 3.5. Small Study Effects (Publication Bias)

As all head-to-head meta-analyses included less than ten studies, publication bias could not be assessed using funnel plots nor formally tested through the Egger regression asymmetry test. In these cases, the available tests for publication are at very high risk of bias because of the lack of statistical power [31].

## 4. Discussion

The main findings of this meta-analysis are the following: (a) all candidate vaccines against chikungunya were able to elicit an immunogenic response in 96% or more of vaccinated subjects, regardless of vaccination schedule and platform used; (b) most vaccines showed the capacity to maintain high seroconversion rates 6 to 12 months after vaccination; (c) four of the five candidate vaccines showed a good overall safety profile (no data were available for ChAdOx1 Chik), with no significant increase in the risk of SAEs among the vaccinated and a low absolute risk of product-related SAEs.

We assessed the immunogenicity and safety of candidate vaccines that were developed based on diverse technology platforms, including live attenuated virus (LAV) (VLA 1553) [16,28,30], viral vectors (including the measles vectored LAV, MV-CHIK [20,22,23,24,29], and the adenovirus vectored presenting subunit particles ChAdOx1 Chik [19]), virus like particles (VLP) (PXVX0317) [18,25,26,27], and m-RNA based vaccine (mRNA-1388) [17], finding no substantial differences across different types of vaccines. All platforms have already received authorization from regulatory agencies for other pathogens, showing both strength and weakness [32]. Typically, LAV vaccines are able to confer an excellent immune response but their potential to revert to a pathogenic virus in people with weakened immune systems has been a cause of concern [13,32,33]. The technology is also labor intensive, demanding strict quality controls for potential biohazards, higher manufacturing costs, and a longer production process, which may be inefficient during epidemic periods [32]. VLP vaccines, on the contrary, theoretically pose less safety concerns compared to LAV but often lead to weaker and shorter responses, needing multiple doses, leading to compliance issues, and the addition of adjuvants [13,32]. Biosafety requirements for manufacturing are less stringent, but difficulties in design, purification, and storage of the vaccines may increase their production costs [32]. Over the last decade, viral vectors have been successfully used for the development of vaccines against Ebola Virus and SARS-CoV-2, showing good immunogenicity, and their easier manufacture and rapid deployment capability make this type of vaccine suitable in the event of epidemics [32]. However, some safety concerns were raised following the report of cases of thrombosis, with thrombocytopenia syndrome (TTS) affecting some population groups after the administration of the viral vector anti-SARS-CoV-2 vaccine [34], which will need to be monitored in the case of chikungunya vaccines. Finally, as regards the mRNA-1388 vaccine, the recently developed mRNA vaccines against SARS-CoV-2 proved to be immunogenic, cost-effective, and relatively easier to manufacture compared to other nucleic acid-based technologies, with the drawbacks of being dependent on ultra-low cold chain transport and being highly reactogenic [32]. Further research is definitely needed to assess mRNA-1388 safety and immunogenicity, as the available data are still limited to a single Phase 1 study with a small sample size.

Concerning the long-term immunogenicity, the available evidence supports the capacity of most candidate vaccines to sustain seroprotection 6 to 12 months after vaccination. No data are available yet on the long-term immunogenicity of the higher concentration (5 × 10⁵ TCID50) of MV-CHIK with the administration of two doses 6 months apart. Riesinger et al. [29] found that the higher dose of MV-CHIK significantly increased concentrations of neutralizing antibodies against CHIKV compared with the lower dose, also in the long term, and that a vaccine boost at 6 months increased antibody titers to a greater extent than the boost at 1 month. However, long-term seroconversion was not assessed for the 6 months boost schedule. Our pooled estimate may thus underestimate the long-term immunogenicity of the vaccine. Evidence is not yet available on the long-term protection conferred by mRNA-1388.

The absolute risk of SAEs following immunization with any of the candidate vaccines was low, and the risk of both all and only product-related SAEs did not significantly increase among the subjects who received any vaccine type. Although the SAEs were not rare (≈1%), our estimates may have been partially increased by the inclusion of some dose-escalation studies, where the number of overall and related adverse events increased with the dose and volume of the candidate vaccine [17,20,26,29]. Additional data are thus needed to precisely estimate the absolute risk of adverse events based on the definitive recommended doses of each vaccine.

As regards the only vaccine that has been licensed by the FDA so far (VLA 1553), our meta-analysis confirms that the vaccine is able to induce a robust and durable immune response in individuals older than 18 years with a single dose, with a satisfactory safety profile, supporting the current indication for use in adult travelers to endemic regions [11]. Some questions still need to be addressed when considering extending vaccination with VLA 1553 to a wider population. First, both pregnant and severely immunocompromised people were excluded from the trials as a precautionary measure, and scarce (n = 59) and non-stratified data were available on the elderly [30], thus information is still needed on the safety of population groups with weakened immune systems, who may be more susceptible to reversion or enhanced replication after the administration of live-attenuated vaccines [13,35]. Second, the available evidence on VLA 1553 is based on data collected in a non-endemic country, and thus the possible effect of pre-existing immunity on the vaccine’s immunogenicity and safety is unknown [30]. Notably, however, the recent technology transfer agreement between Valneva ^®^ and the Instituto Butantan in Brazil, in order to manufacture and supply VLA 1553 to the surrounding regions, may facilitate an equitable distribution of the vaccine to resource-limited settings and facilitate the assessment of the vaccine effectiveness and safety in the populations of endemic regions [36].

Besides VLA 1553, the other vaccines analyzed in this review still require further steps to obtain the marketing authorizations from drug regulatory agencies. As regards their current schedule to licensure, a phase III trial for PXVX0317 has been completed and the results are currently under quality control on ClinicalTrials.gov [37]. MV-CHIK received an FDA fast track designation after the positive evaluation of the results of the phase II trial [38], while we could not find information of planned or on-going phase II trials for mRNA-1388 and ChAdOx1 Chik vaccine candidates.

This meta-analysis has some limitations that must be considered in interpreting the results. First, no data are available on the real-word effectiveness of the candidate vaccines, and although they all showed an excellent immunogenicity, which is known to be a very good proxy of efficacy [21], post-marketing trials or observational longitudinal analyses from endemic countries will be needed to confirm the present promising findings. Second, as already stated, we found no data on frail populations, who show a higher risk of developing severe disease [13,35], and there is an urgent need for future studies on children, elderly, and/or immunocompromised subjects. Finally, nearly all the included studies were pre-market RCTs sponsored by the manufacturers, and it will thus be important to obtain additional data from independent studies.

## 5. Conclusions

All chikungunya vaccine candidates showed a good safety profile and an excellent immunogenicity, which remained stable 6–12 months after the primary vaccination course for most vaccines. These findings support the potential use of the vaccines for the prevention of chikungunya and the current indication for use in adult travelers to endemic regions of the only vaccine that has been licensed so far (VLA 1553). In a view to extend chikungunya vaccination to a wider audience and halt the spread of CHIKV, future studies are needed on the vaccine safety and efficacy in individuals with pre-existing immunity from endemic countries as well as high-risk populations such as children, the elderly, and people with comorbidities.

## Figures and Tables

**Table 1:** Characteristics of the included studies.

First Author	CT.Gov ID	Year	Vaccine Type	Age, Mean (y)	% F	Country	Design	Blinding	Doses ^A^	Control	Outcome(s) Extracted	Sponsor
Folegatti [19]	NCT03590392	2021	ChAdOx1 Chik	18–50; 29.0	75.0	UK	Single-arm	No	1	No, dose finding	I; S	GTRG (OxfordUniversity)
Ramsauer [20]	EUDRACT 2013-001084-23	2015	MV-CHIK	18–45; 31.0	52.0	Austria	RCT ^B^	Double	2, 28 days apart	MMR vaccine	I; S	Themis Bioscience (T-Bio)
Reisinger [29]	NCT02861586	2018	MV-CHIK	18–55; 29.5	47.0	Austria, Germany	RCT	Double	2, 28 days apart	Placebo and MMR vaccine	I; S	T-Bio
NCT03101111 [22]	NCT03101111	2019	MV-CHIK	21–50; 33.2	41.0	Puerto Rico	RCT	Double	2, 28 days apart	Placebo and MMR vaccine	S	T-Bio
NCT03635086 [23]	NCT03635086	2019	MV-CHIK	18–55; 36.9	38.3	UK	Single-arm ^C^	Double	2, 28 days apart	No ^C^	S	T-Bio
NCT03807843 [24]	NCT03807843	2021	MV-CHIK	21–65; 39.3	61.0	Puerto Rico	RCT	Double	2, 28 days apart	Placebo	S	T-Bio
Shaw [17]	NCT03325075	2023	mRNA-1388	18–49; 31.6	56.7	USA	RCT	Single	2, 28 days apart	Placebo	I; S	Moderna
Wressnigg [16]	NCT03382964	2020	VLA1553	18–45; 32.3	23.0	USA	Single-arm ^C^	Single	1	No ^C^	I; S	Valneva
Schneider [30]	NCT04546724	2023	VLA1553	≥18; 45.0	54.7	USA	RCT	Double	1	Placebo	I; S	Valneva
McMahon [28]	NCT04786444	2024	VLA1553	18–45; 33.2	54.7	USA	Single-arm ^C^	Double	1	No control	I; S	Valneva
Chang [18]	NCT01489358	2014	PXVX0317	18–50; 31.0	60.0	USA	Single-arm	No	2, 28 days apart	No ^C^	I; S	NIH
Chen [27]	NCT02562482	2020	PXVX0317	18–60; 35.0	50.0	Several	RCT	Double	2, 28 days apart	Placebo	I; S	NIH
Bennett [26]	NCT03483961	2022	PXVX0317	18–45; 32.0	60.0	USA	Single-arm ^C^	Double	2, 28 days apart	No ^C^	I; S	Emergent Biosolutions (EB)
NCT05065983 [25]	NCT05065983	2019	PXVX0317	18–45; 31.0	56.0	USA	Single-arm	No	1	No control	I; S	EB

PXVX0317 vaccine is also defined as VRC-CHKVLP059-00-VP; mRNA-1388 vaccine is also defined VAL-181388. F = Females; RCT = Randomized Controlled Trial; SAEs = Serious Adverse Events; I = Immunogenicity outcomes (seroresponse/seroconversion rate); S = Safety outcomes (e.g., incidence of SAEs); y = years; MMR = Measles, Mumps, and Rubella vaccine. ^A^ Referred to the extracted data, which may not coincide with the overall number of doses that were administered during the trial. ^B^ Single-arm trial for immunogenicity, RCT for SAEs. ^C^ Dose-finding trial; the results on the immunogenicity and SAEs were analyzed as a single-arm trial.

**Table 2:** Rates of seroconversion/seroresponse ^A^ and serious adverse events following the administration of various Chikungunya vaccines. Data from single studies have been combined using proportion meta-analysis (random-effect model, PP data).

	Short-Term Seroconversion ^B^		Long-Term Seroconversion ^C^	
	Sample (n/N)	Seroconversion ^A^,% (95% CI)	Sample (n/N)	Seroconversion ^A^,% (95% CI)
All vaccines	1420/1441	99.6 (98.5–100)	1080/1131	96.7 (91.3–99.8)
Vaccine type (n. of doses)				
− MV-CHIK (2)	191/200	96.5 (93.1–99.0)	66/93	71.0 (61.1–79.2)
− VLA1553 (1)	645/656	98.8 (97.6–99.6)	608/627	97.9 (95.6–99.5)
− PXVX0317/VRC-CHKVLP059-00-VP (2)	517/518	100 (99.8–100)	382/387	99.0 (97.6–99.8)
− Others: ChAdOx1 Chik (1), and mRNA-1388/VAL-181388 (2)	67/67	100 (97.3–100)	24/24	100 (86.2–100)
Vaccine doses				
− 1 dose	694/705	99.2 (98.2–99.9)	632/651	98.2 (96.3–99.6)
− 2 doses	726/736	99.7 (97.5–100)	448/480	93.1 (73.7–100)
	**Any SAE** ^D^		**Product-related SAE** ^E^	
	Sample (n/N)	‰ (95% CI)	Sample (n/N)	‰ (95% CI)
All vaccines	69/4480	6.9 (3.9–10.5)	4/4392	0.0 (0.0–0.0)
Vaccine type (n. of doses)				
− MV-CHIK (2)	6/353	8.3 (0.1–24.4)	1/265	0.1 (0.0–10.6)
− VLA1553 (1)	52/3520	9.8 (6.2–14.0)	2/3520	0.0 (0.0–0.0)
− PXVX0317/VRC-CHKVLP059-00-VP (2)	10/540	11.7 (2.5–25.1)	0/540	0.0 (0.0–0.1)
− Others: ChAdOx1 Chik (1), and mRNA-1388/VAL-181388 (2)	1/67	10.5 (0.0–59.2)	1/67	10.5 (0.0–59.2)
Vaccine doses				
− 1 dose	52/3569	5.8 (2.7–9.7)	2/3569	0.0 (0.0–0.0)
− 2 doses	17/911	11.5 (4.0–21.5)	2/823	0.0 (0.0–1.1)

CI = Confidence Interval. PP = Per-protocol. n/N = number of cases/total sample; SAEs = Serious Adverse Events. ^A^ Seroresponse/seroconversion rates were extracted from single studies following the definition provided by the authors. When available, seroconversion rates have been extracted; ^B^ 28 days after vaccination; ^C^ 6 or 12 months after vaccination. When both were available, 12-month data have been extracted; ^D^ All serious adverse events; ^E^ Product-related serious adverse events only.

**Table 3:** Results of head-to-head meta-analyses on serious adverse events (SAEs) of Chikungunya vaccines versus controls according to vaccine type (random-effect model, PP data).

	Any SAE ^A^		Product-Related-SAE ^B^	
	Datasets (N)	Pooled RR (95% CI)	Datasets (N)	Pooled RR (95% CI)
All vaccines	7 (4898)	0.80 (0.38–1.69)	7 (4867)	0.98 (0.37–2.59)
Vaccine type				
− MV-CHIK	4 (362)	0.57 (0.19–1.70)	4 (331)	0.89 (0.27–2.88)
− VLA1553	1 (4115)	1.93 (0.91–4.07)	1 (4115)	1.68 (0.08–34.9)
− PXVX0317 (or VRC-CHKVLP059-00-VP)	1 (363)	0.34 (0.11–1.03)	1 (363)	1.02 (0.06–16.1)
− Others (ChAdOx1 Chik or mRNA-1388)	1 (58)	1.09 (0.05–25.4)	1 (58)	1.09 (0.05–25.4)

RR = Risk Ratio; 95% CI = Confidence Intervals. ^A^ All serious adverse events, either related or unrelated to the administered product. ^B^ Product-related serious adverse events only.

## Data Availability

All data are available from the studies that have been included in the meta-analysis.

## Supplementary Materials

The following supporting information can be downloaded at: https://www.mdpi.com/article/10.3390/vaccines12090969/s1, Table S1: List of articles excluded after the full-text screening process and reasons of exclusion; Table S2: Vaccine composition and methodology adopted to define immune response in each included study. Table S3: Risk of bias of the included studies, assessed using the revised Cochrane risk-of-bias tool for randomized trials; Figure S1: Pooled seroconversion/seroresponse rates 28 days after Chikungunya vaccination, by vaccine type (A) and number of doses (B); Figure S2: Pooled seroconversion/seroresponse rates 6–12 months after Chikungunya vaccination, by vaccine type (A) and number of doses (B); Figure S3: Pooled rates of any serious adverse event after Chikungunya vaccination, by vaccine type (A) and number of doses (B); Figure S4: Pooled rates of product-related serious adverse events after Chikungunya vaccination, by vaccine type (A) and number of doses (B).
