# Supplementary material for: Immunogenicity and Safety of Chikungunya Vaccines: A Systematic Review and Meta-Analysis
Source: Vaccines (Basel). 2024 Aug 27;12(9):969. doi: 10.3390/vaccines12090969 (PMC11436237; doi:10.3390/vaccines12090969)
Supplement: Supplementary file 1 [file vaccines-12-00969-s001.zip › vaccines-3121423-supplementary.pdf]

SUPPLEMENTARY MATERIAL

Table S1. List of the excluded studies after the full-text screening process, and reasons of exclusion.

Reference	Trial ID	Reason for exclusion
Raju S, et al. A chikungunya virus-like particle vaccine induces broadly neutralizing and protective antibodies against alphaviruses in humans. Sci Transl Med. 2023;15(696):eade8273.	NCT03483961	Same population of Bennett 2022 [1]; no additional data could be extracted.
Tschismarov R, et al. Antibody effector analysis of prime versus prime-boost immunizations with a recombinant measles-vectored chikungunya virus vaccine. JCI Insight 2021;6(21):e151095.	NCT02861586	Same population of Reisinger 2019 [2]; no additional data could be extracted.
Study to demonstrate consistency of three lots of live-attenuated chikungunya virus vaccine candidate in healthy adults. 2022, ClinicalTrials.gov	NCT04786444	Same population of McMahon 2024 [3]; no additional data could be extracted.
Pivotal study to evaluate safety and immunogenicity of live-attenuated chikungunya virus vaccine candidate in adults. 2021, ClinicalTrials.gov	NCT04546724	Same population of Shaw 2023 [4]; no additional data could be extracted.
Safety, tolerability and immunogenicity of VAL-181388 in healthy participants. 2019, ClinicalTrials.gov	NCT03325075	Same population of Schneider 2023 [5]; no additional data could be extracted.

Table S2: Vaccine composition Methodology adopted to define immune response in each included study.

First author	Vaccine type	Vaccine composition	Immunogenicity measurement
Folegatti [6]	ChAdOx1 Chik	ChAdOx1 Chik is a chimpanzee adenoviral vector vaccine expressing the CHIKV structural proteins: Capsid, E1, E2, E3 and 6K.<br>The vaccine was based on multiple CHIKV lineages: CHIKV-LR (Indian Ocean Lineage, IOL), SV-0444 (Asian Lineage), 37997 (West African Lineage), and YO111213 (Asian/American Lineage).	Induction of serum neutralizing antibodies was evaluated with a PRNT on monolayers of Vero cells (Vero ATCC CCL-81) cultured in 12-well plates. Total anti-CHIKV IgG (GMT) was measured using a standardized in-house indirect ELISA.<br>Titers were quantified as the highest serum dilution that inhibited plaque formation in 50% (PRNT50). Seroconversion was considered positive in samples with reciprocal titers of PRNT50 ≥10.
Ramsauer [7]<br>Reisinger [2]<br>NCT03101111 [8]<br>NCT03635086 [9]<br>NCT03807843 [10]	MV-CHIK	The MV-CHIK vaccine is a measles-vectored vaccine based on the Schwarz vaccine strain. A chikungunya virus (La Reunion strain 06-46) subgenomic open reading frame, encoding for structural genes Capsid, E1, E2, E3 and 6K, is introduced into the measles virus vector.	The presence of serum neutralising antibodies was determined with PRNT on Vero-cell monolayers (Vero ATCC CCL-81) in six-well plates, shown by a reduction in the numbers of input virus plaques by at least 50% (PRNT50).<br>As a secondary endpoint, the authors measured the GMT using an ELISA.<br>The authors used hemagglutination inhibition assays according to the method of Clarke and Casals with a South African strain of chikungunya virus at a concentration of 4 hemagglutinating units. Seroconversion was defined as the participant having a neutralising antibody titre of 10 or greater (i.e., a 1/10 or greater dilution of the participant's serum giving a positive PRNT50 result).<br>Immunogenicity data could not be extracted from NCT03101111 [8], NCT03635086 [9], NCT03807843 [10].
Shaw [4]	mRNA-1388 / VAL-181388	The mRNA-1388 (also known as VAL-181388) vaccine comprises chemically modified mRNA encoding the full CHIKV structural polyprotein (Capsid and envelope proteins E1, E2, E3, and 6k/TF) from CHIKV Senegal strain 37,997 (West African genotype) and a lipid nanoparticle (LNP) delivery. system.	Serum antibody neutralization titers were measured using a plaque-reduction neutralization test EC50 (PRNT50). CHIKV (West African strain 37997) was mixed with heat-inactivated, 2-fold serial dilutions of serum (37 °C for 1 h) and added to Vero 76 cell monolayers in 12-well plates.<br>GMT were measured by ELISA.<br>Seroconversion was considered positive in samples with reciprocal titers of PRNT50 ≥10.
Wressnigg [11]<br>Schneider [5]<br>McMahon [3]	VLA1553	The vaccine was based on La Reunion strain (East Central South African genotype).	Immune response after vaccination was measured for chikungunya virus-specific neutralising antibodies and assessed by use of a validated micro plaque reduction neutralisation test (μPRNT) with a heterologous chikungunya virus strain (TSI-GSD-218 strain).<br>Equal volumes of serial two-fold dilutions of serum samples were mixed with VLA1553 and incubated for 60–100 min at 37°C, before transfer onto Vero cells plated in 96-well plates. After 5 days, inhibition of infection was observed by assessing cell viability.<br>Seroconversion for baseline positive participants was defined as a more than 4-fold increase over baseline. Seroprotection was defined as μPRNT50 150 or more.14 Negative samples with a μPRNT50 titre less than 20 were imputed with 10.<br>GMT were measured by an IgG CHIKV-specific ELISA Euroimmun)
Chang [12]<br>Chen [13]<br>Bennett [1]<br>NCT05065983 [14]	PXVX0317 / VRC-CHKVLP059-00-VP	The vaccine is an investigational VLP vaccine that consists of the E1, E2 and capsid proteins of CHIKV Senegal strain 37997 (West African genotype).	The CHIKV-specific neutralizing antibody responses were measured using the attenuated CHIKV strain 181/cone 25 in a focus reduction neutralization test and reported as effective concentration (EC50) values. The EC50 is the dilution of sera that inhibits 50% infection in the viral neutralization assay. Durability of the immune response was measured in a different laboratory using a CHIKV luciferase neutralization assay through 72 weeks for vaccine recipients found to be seronegative at baseline and reported as NT80 values. NT80 is the reciprocal dilution of sera associated with an 80% reduction in viral activity. GMT were measured by antibody assays that include antigen-specific ELISA.

CHIKV = Chikungunya Vaccine. GMT = Geometric Mean Titers. ELISA = Enzyme-Linked ImmunoSorbent Assay. PRNT = Plaque Reduction Neutralization Tests.

Table S3. Risk of bias of the included studies, assessed using the revised Cochrane risk-of-bias tool for randomized trials.

First author	Randomization process	Deviations from Intended interventions	Missing Outcome data	Measurement of the outcome	Selection of reported results	Overall bias
Chang [12] *	Some concerns	Low risk	Low risk	Some concerns	Low risk	Low risk
Ramsauer [7]	Low risk	Low risk	Low risk	Low risk	Low risk	Low risk
Reisinger [2]	Low risk	Low risk	Low risk	Low risk	Low risk	Low risk
NCT03807843 [10]	Low risk	Low risk	Low risk	Low risk	Low risk	Low risk
NCT03635086 [9]	Low risk	Low risk	Low risk	Low risk	Low risk	Low risk
NCT03101111 [8]	Low risk	Low risk	Low risk	Low risk	Low risk	Low risk
Chen [13]	Low risk	Low risk	Low risk	Low risk	Low risk	Low risk
Wressnigg [11]	Low risk	Low risk	Low risk	Low risk	Low risk	Low risk
NCT05065983 [15] *	Some concerns	Low risk	Low risk	Some concerns	Low risk	Low risk
Folegatti [6] *	Some concerns	Low risk	Low risk	Some concerns	Low risk	Low risk
Bennett [1]	Low risk	Low risk	Low risk	Low risk	Low risk	Low risk
Shaw [4]	Low risk	Low risk	Low risk	Low risk	Low risk	Low risk
Schneider [5]	Low risk	Low risk	Low risk	Low risk	Low risk	Low risk
McMahon [3]	Low risk	Low risk	Low risk	Low risk	Low risk	Low risk

* Trial not randomized.

Figure S1. Pooled seroconversion / seroresponse rates 28 days after Chikungunya vaccination, by vaccine type (A) and number of doses (B).

A

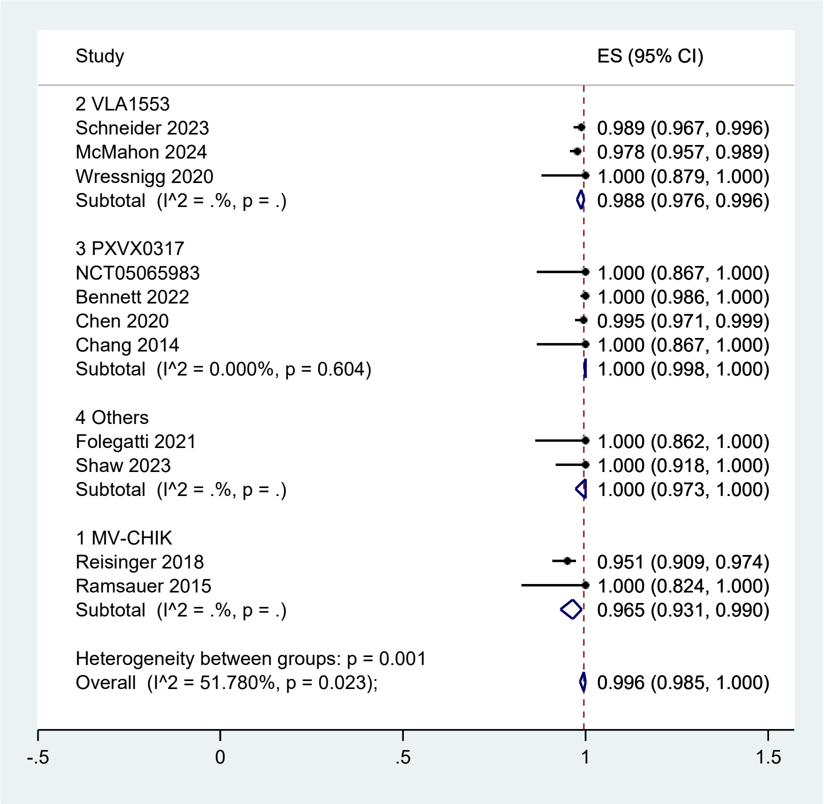

B

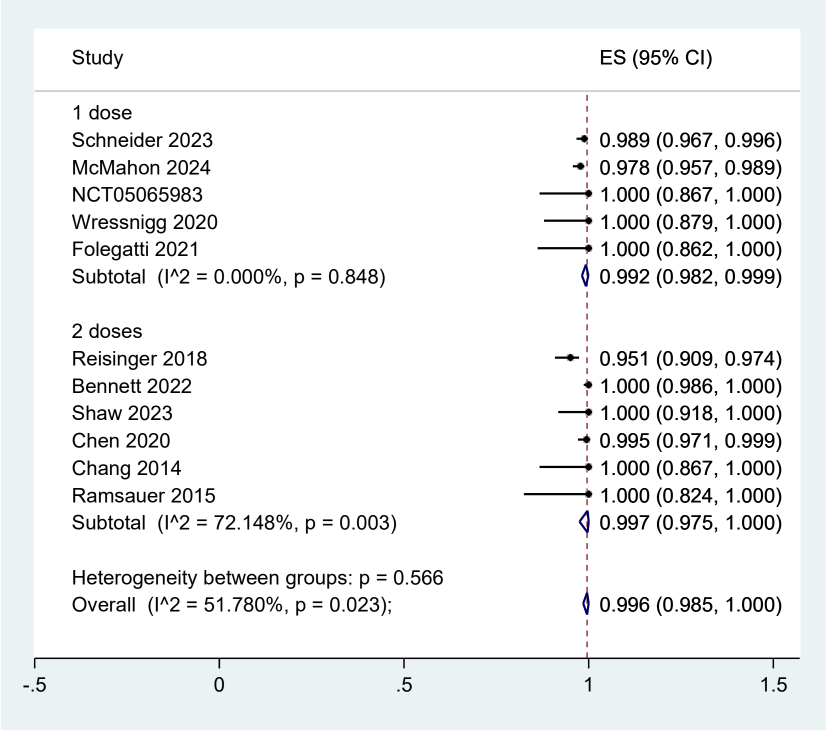

Figure S2. Pooled seroconversion / seroresponse rates 6-12 months after Chikungunya vaccination, by vaccine type (A) and number of doses (B).

A

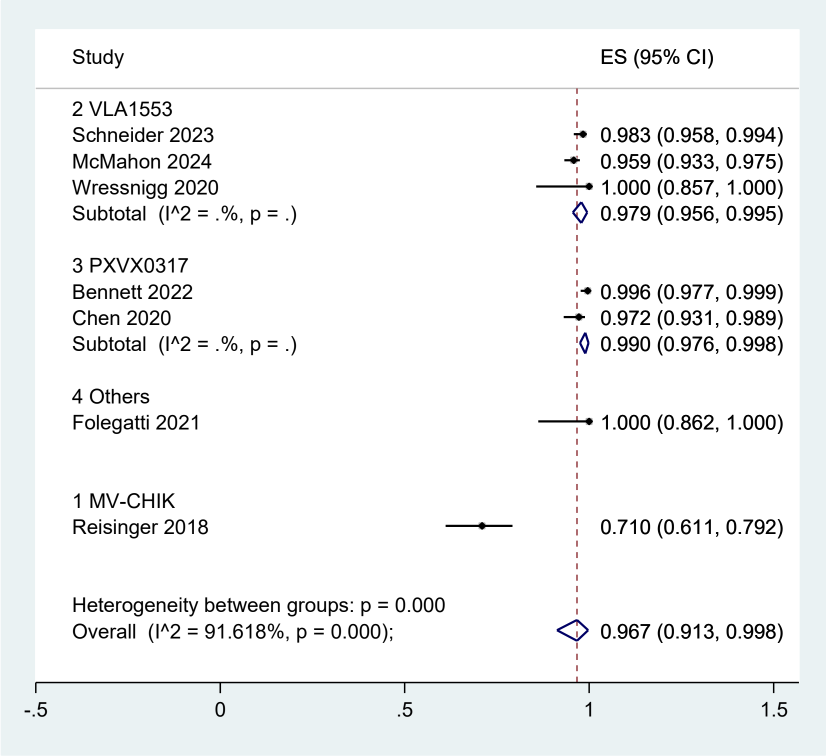

B

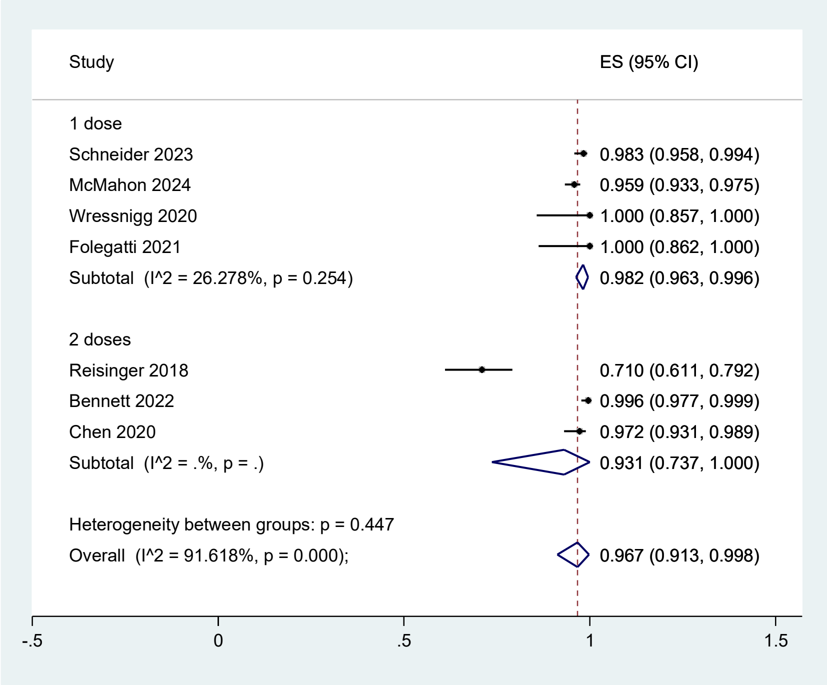

Figure S3. Pooled rates of any serious adverse event after Chikungunya vaccination, by vaccine type (A) and number of doses (B).

A

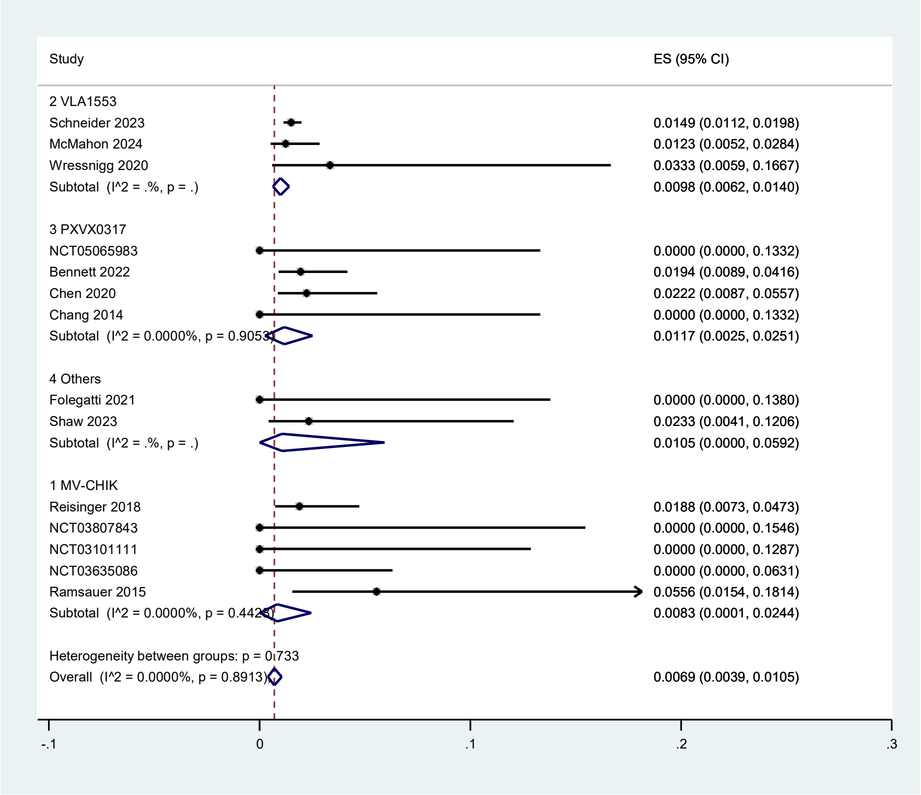

B

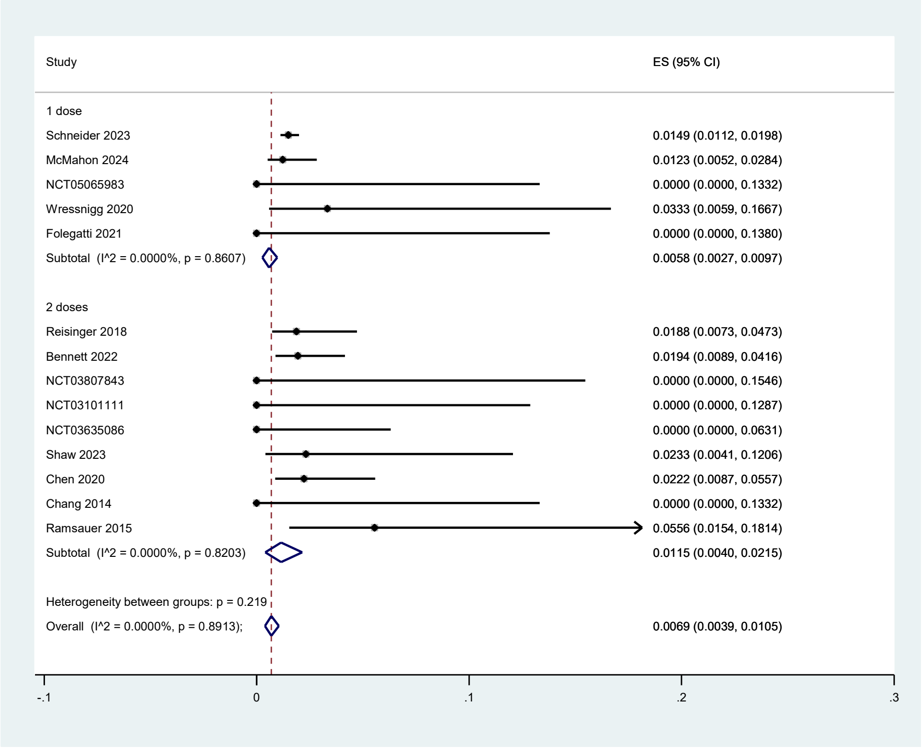

Figure S4. Pooled rates of product-related serious adverse events after Chikungunya vaccination, by vaccine type (A) and number of doses (B).

A

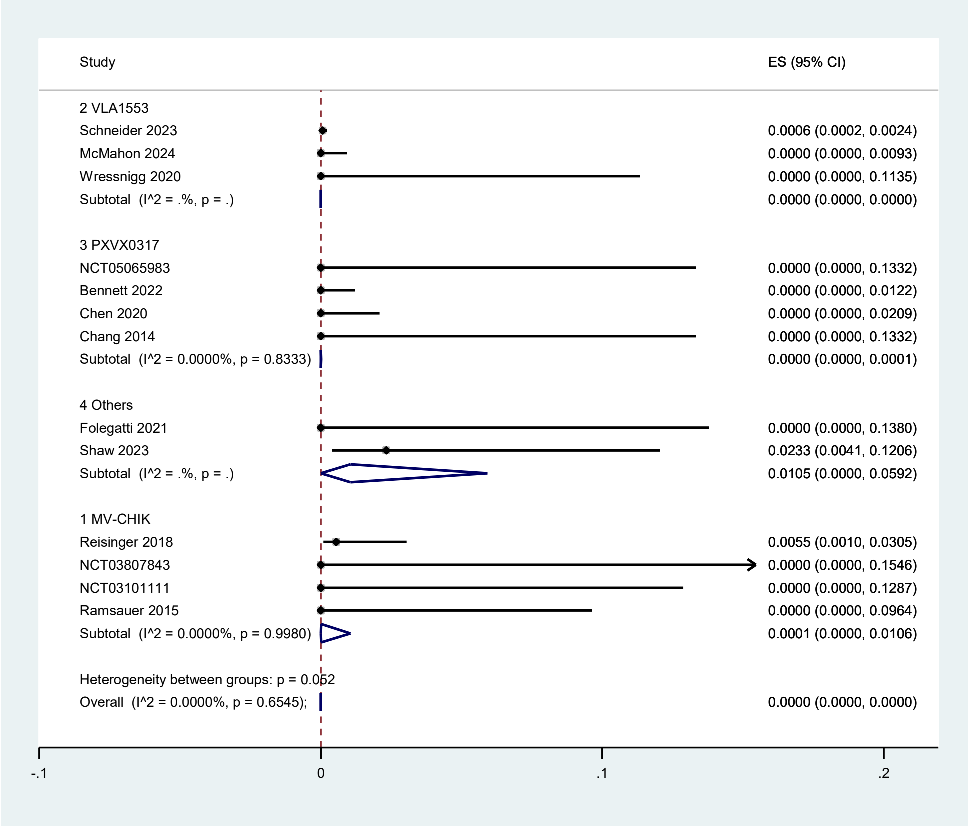

B

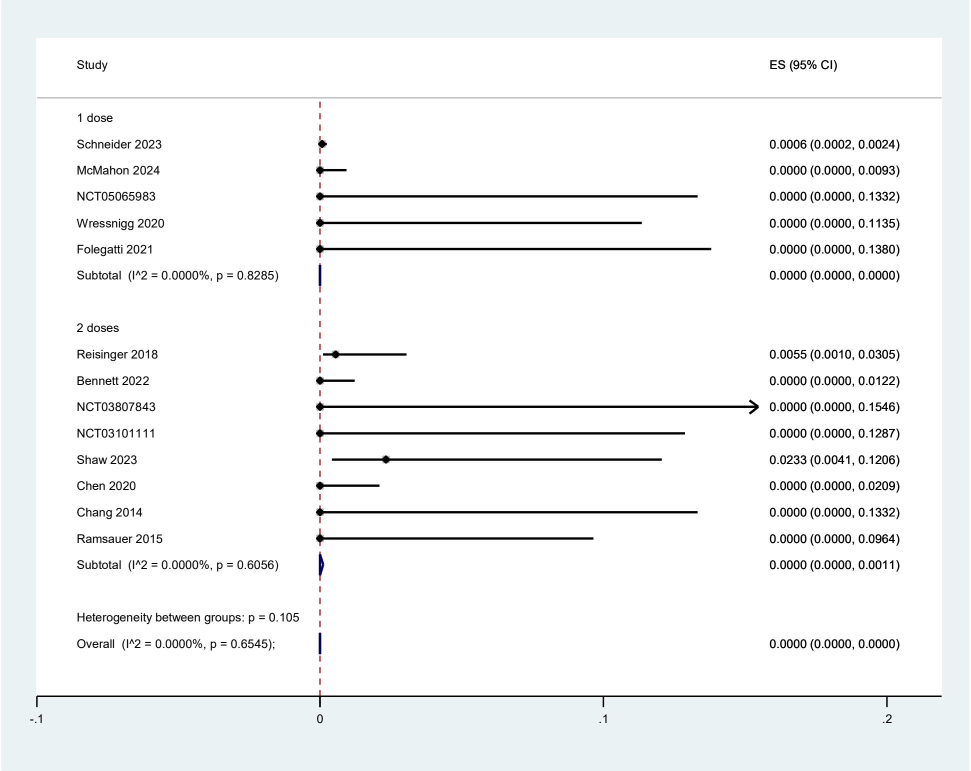

References

1. Bennett, S.R., et al., *Safety and immunogenicity of PXVX0317, an aluminium hydroxide-adjuvanted chikungunya virus-like particle vaccine: a randomised, double-blind, parallel-group, phase 2 trial.* Lancet Infect Dis, 2022. **22**(9): p. 1343-1355.
2. Reisinger, E.C., et al., *Immunogenicity, safety, and tolerability of the measles-vectored chikungunya virus vaccine MV-CHIK: a double-blind, randomised, placebo-controlled and active-controlled phase 2 trial.* Lancet, 2019. **392**(10165): p. 2718-2727.
3. McMahon, R., et al., *A randomized, double-blinded Phase 3 study to demonstrate lot-to-lot consistency and to confirm immunogenicity and safety of the live-attenuated chikungunya virus vaccine candidate VLA1553 in healthy adultsdagger.* J Travel Med, 2024. **31**(2).
4. Shaw, C.A., et al., *A phase 1, randomized, placebo-controlled, dose-ranging study to evaluate the safety and immunogenicity of an mRNA-based chikungunya virus vaccine in healthy adults.* Vaccine, 2023. **41**(26): p. 3898-3906.
5. Schneider, M., et al., *Safety and immunogenicity of a single-shot live-attenuated chikungunya vaccine: a double-blind, multicentre, randomised, placebo-controlled, phase 3 trial.* Lancet, 2023. **401**(10394): p. 2138-2147.
6. Folegatti, P.M., et al., *A single dose of ChAdOx1 Chik vaccine induces neutralizing antibodies against four chikungunya virus lineages in a phase 1 clinical trial.* Nat Commun, 2021. **12**(1): p. 4636.
7. Ramsauer, K., et al., *Immunogenicity, safety, and tolerability of a recombinant measles-virus-based chikungunya vaccine: a randomised, double-blind, placebo-controlled, active-comparator, first-in-man trial.* Lancet Infect Dis, 2015. **15**(5): p. 519-27.
8. NCT03101111, *Study of a Live Attenuated Chikungunya Vaccine in a Previously Epidemic Area*. 2019, ClinicalTrials.gov.
9. NCT03635086, *Safety, Tolerability and Long-term Immunogenicity of Different Formulations of a Chikungunya Vaccine (V184-005)*. 2011, ClinicalTrials.gov.
10. NCT03807843, *Chikungunya Vaccine (V184) Study in Previously Exposed Adults (V184-006)*. 2019, ClinicalTrials.gov.
11. Wressnigg, N., et al., *Single-shot live-attenuated chikungunya vaccine in healthy adults: a phase 1, randomised controlled trial.* Lancet Infect Dis, 2020. **20**(10): p. 1193-1203.
12. Chang, L.J., et al., *Safety and tolerability of chikungunya virus-like particle vaccine in healthy adults: a phase 1 dose-escalation trial.* Lancet, 2014. **384**(9959): p. 2046-52.
13. Chen, G.L., et al., *Effect of a Chikungunya Virus-Like Particle Vaccine on Safety and Tolerability Outcomes: A Randomized Clinical Trial.* JAMA, 2020. **323**(14): p. 1369-1377.
14. NCT05065983, *A Study to Assess the Safety and Immunogenicity of PXVX0317 Chikungunya Virus Virus-Like Particle Vaccine (CHIKV VLP)*. 2019, ClinicalTrials.gov.
15. NCT05065983, *A Study to Assess the Safety and Immunogenicity of PXVX0317 Chikungunya Virus Virus-Like Particle Vaccine (CHIKV VLP)*. 2021, ClinicalTrials.gov.
